# Acoustic Changes in Voicemail Transmission Induced by Mobile Phones

**DOI:** 10.1055/s-0045-1810029

**Published:** 2025-10-16

**Authors:** Halil Erdem Özel

**Affiliations:** 1 Department of Otolaryngology, University of Health Sciences Derince Research and Training Hospital, Kocaeli, Turkey

**Keywords:** voice, voice quality, mobile phone

## Abstract

**Introduction:**

Given the widespread use of mobile phones for voice communication, a comprehensive understanding of the objective acoustic alterations in voicemail transmission is lacking, motivating this investigation into voice quality changes.

**Objective:**

This study aims to explore the objective changes in voice recordings caused by mobile phones and voicemail applications during the transmission process, specifically focusing on the alterations to voice quality that occur during this process.

**Methods:**

A volunteer sample of 45 healthy male hospital employees, with an average age of 36.7 ± 7.5 (ranging from 22 to 50), in a tertiary referral center were included in this study. The Multi-Dimensional Voice Program (Kay Elemetrics, Lincoln Park, NJ, USA) was employed to compare a set of nine parameters derived from sustained vowel phonations of /a/, encompassing free-field voice and mobile phone voicemail recordings. Average fundamental frequency (Fo), frequency perturbation parameters [Pitch Period Perturbation Quotient (PPQ), Relative Average Perturbation (RAP)], amplitude perturbation parameters [Shimmer in dB (ShdB), Shimmer Percent (Shim), Amplitude Perturbation Quotient (APQ)], noise parameters [Noise-to-Harmonic Ratio (NHR)] were calculated.

**Results:**

Analysis of the patient data revealed that fundamental frequency (Fo) was resistant to alterations of voice ( *p* = 0,313). Frequency perturbation parameters (PPQ, RAP) were impacted ( *p* = 0.018, 0.020 respectively), however, amplitude perturbation parameters (ShdB, Shim, APQ) and noise parameter (NHR) were much more affected in voice transmission caused by mobile phones ( *p* < 0,001 in all).

**Conclusion:**

The findings of this study indicate that mobile phones induce significant acoustic changes in voicemail transmission. The fundamental frequency remained resistant to alterations in voice.

## Introduction

Mobile phones have become an integral aspect of contemporary social life. The inability to use a phone can significantly impact daily living, potentially restricting job opportunities. Communication via phone poses a unique challenge for individuals with hearing loss, including those with cochlear implants and traditional hearing aids. 1 2 However, there is a scarcity of literature investigating the specific reasons for this challenge.

Voice interactions and voice messaging on mobile phones are experiencing rapid growth in popularity. Consequently, there is a rising emphasis on enhancing voice message technology. 3 Some studies have suggested that wireless connections between phones and hearing aids can enhance speech intelligibility. 4 5 6 7 In studies examining the acoustic quality of mobile phone voice recordings, some authors have asserted that smartphones' measurements of acoustic voice parameters are reliable in clinical settings, while others have contested this viewpoint. 8 9 10 Studies demonstrate that sound frequencies can be reliably measured using mobile phone applications. 11 The contradictory results in these studies may stem from methodological differences, such as the type of recording devices used, the recording environments, and the analysis techniques. Some studies employed high-quality clinical microphones, while others used smartphone microphones or mobile communication devices. The recording conditions varied, with some conducted in anechoic chambers and others in standard clinical or noisy environments. Additionally, the inclusion of onset/offset segments, different software for acoustic analysis, and variations in participant populations may have contributed to discrepancies in results. Notably, these studies have not delved into the transmission of voice to the receiving party. However, the exact way voicemail apps process and transmit sound remains unclear, potentially due to their specific coding methods.

Research has shown that telephone signal transmission significantly alters the acoustic properties of voice, affecting both objective measurements and perceptual evaluations. Commonly observed issues include amplitude distortion and frequency response modifications, which impact speech clarity and overall quality. 12 13 Interestingly, the mean fundamental frequency remains relatively stable across different telepractice platforms. 14 Additionally, perceptual assessments of voice quality are influenced by telephone transmission. 13 15 As a result, telephone transmission introduces various acoustic distortions that must be carefully considered, especially in applications like forensic analysis, where precise voice assessment is crucial. Despite existing research highlighting significant changes in voice transmitted over the telephone, studies specifically examining objective alterations remain limited.

This study aims to explore the objective changes in voice recordings caused by mobile phones and voicemail applications during the transmission process, specifically focusing on the alterations to voice quality that occur during this process.

## Methods

This study was performed at a tertiary health center. The participants comprised 45 volunteer health workers with an average age of 36.7 ± 7.5 (mean ± standard deviation). All volunteers were male, and their ages ranged between 22 and 50 years to ensure standardization. Individuals with acid reflux, asthma, allergies, acute respiratory infections, as well as those who had undergone laryngeal or thyroid surgery, were excluded from the study. Perceptual voice evaluations were conducted on the volunteers before the test, and only participants with healthy voices were included in the study. These evaluations were performed by an otolaryngologist using the GRBAS scale. Participants were considered to have 'healthy voices' if they received a global severity rating of 0 (normal) on the GRBAS scale.

Acoustic measures were compared between voice signals simultaneously recorded from healthy speakers through an iPhone (iPhone 6s model A1303, Apple, USA) and a recording system with a microphone (Shure SM58; Shure Inc., Niles, IL). The recording was performed using a laptop computer with a sampling frequency of 44,100 kHz and a resolution of 16 bits in '.nsp' format via the Multi-Dimensional Voice Program (MDVP; Kay Elemetrics, Lincoln Park, NJ, USA). The mobile phone was placed adjacent to the microphone with a holder. Voice samples were recorded at ∼10 cm from the lips, directed toward the mouth at a 45-degree angle, in a soundproof and anechoic room. The speakers were instructed to produce a steady sustained phonation of the vowel /a/ for ∼5 seconds at habitual loudness and pitch. Voice breaks were not present in the analyzed segments considered for evaluation. Three phonations were then recorded, and the one with the best voice analysis measurement was selected to ensure that any observed changes were due to mobile phone transmission rather than variability in the original voice production. The initial and final 20 cycles of each phonation were excluded from the analysis to avoid perturbations related to the onset and offset of phonation.

Voice recordings through the mobile phone were conducted using the WhatsApp voice messaging application (WhatsApp Inc., Attn: WhatsApp Copyright Agent, 1601 Willow Road, Menlo Park, California 94025, USA) and sent to another WhatsApp and iPhone 6s user. Voicemail recordings were analyzed instead of direct phone call voices to eliminate voice transmission problems associated with telephone networks. To minimize factors affecting the acoustic quality of WhatsApp voice messages identical devices and WhatsApp versions were used. The primary focus of the study was to examine the changes in voice caused by the mobile phone and voicemail application. Mobile phone recordings in 'mp4' format were transferred to a Mac computer, and via iTunes software (Apple Inc.), 'p4' files were converted to '.wav' files to enable analysis with MDVP. The same duration of voice recording segments was utilized for comparing the free-field and mobile phone voices.

After voice recordings of all patients were completed, all voice samples were analyzed with MDVP. Seven clinically significant parameters, which can be grouped under four headings, were selected for analysis:

1. Fundamental frequency parameters:

Average fundamental frequency (Fo) /Hz/: Average value of all extracted period-to-period fundamental frequency values.

2. Frequency perturbation parameters:

Pitch period perturbation quotient (PPQ) /%/: Relative evaluation of the period-to-period variability of the pitch within the analyzed voice sample with a smoothing factor of 5 periods.

Relative average perturbation (RAP) /%/: Relative evaluation of the period-to-period variability of the pitch within the analyzed voice sample with a smoothing factor of 3 periods.

3. Amplitude perturbation parameters:

Shimmer in dB (ShdB) /dB/: Evaluation in dB of the period-to-period (very short-term) variability of the peak-to-peak amplitude within the analyzed voice sample.

Shimmer percent (Shim)/%/: Relative evaluation of the period-to-period (very short-term) variability of the peak-to-peak amplitude within the analyzed voice sample.

Amplitude perturbation quotient (APQ) /%/: Relative evaluation of the period-to-period variability of the peak-to-peak amplitude within the analyzed voice sample at the smoothing level of 11 periods.

4. Noise parameters:

Noise-to-Harmonic Ratio (NHR): Average ratio of the inharmonic spectral energy in the frequency range 70–4200 Hz to the harmonic spectral energy in the frequency range 70–4200 Hz. This is a general evaluation of the noise present in the analyzed signal.

The data were analyzed using the software Statistical Product and Service Solutions (SPSS), Predictive Analytics Software (PASW), and Statistics 21 (SPSS Inc.; IBM Corp., Chicago, IL, USA). A paired student's *t* -test was applied to the measurements in all groups. A p-value of <0.05 was considered statistically significant.

## Results

**Fig. 1:**
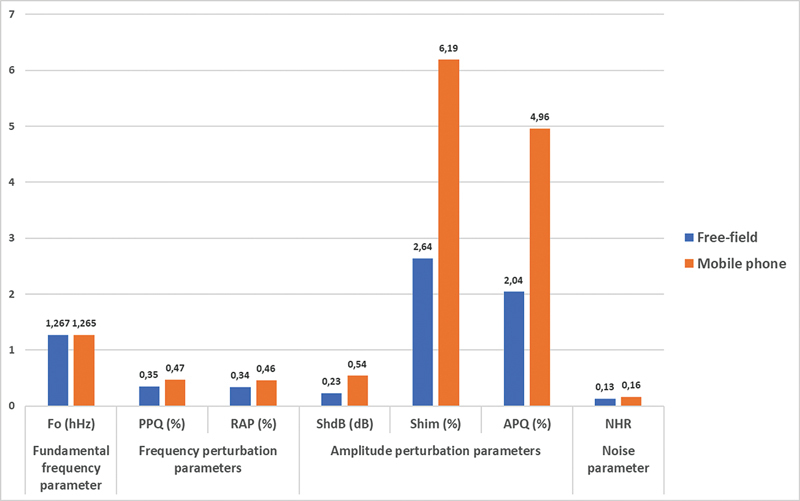
Comparison of acoustic measurements of free field and mobile phone voicemail recordings.

Analysis of the patient voice data, comparing the free-field voice and the mobile phone recordings, revealed that Fo is resistant to alterations in voice ( *p* = 0.313). Frequency perturbation parameters (PPQ, RAP) were affected ( *p* = 0.018, 0.020, respectively); however, amplitude perturbation parameters (ShdB, Shim, APQ) and the noise parameter (NHR) were significantly impacted in voice transmission caused by mobile phones ( *p* < 0.001 in all). The mean and standard deviation of acoustic measures for the free-field and the mobile phone voicemail recordings, along with p values, are summarized in Table 1 . A comparison of acoustic measurements between free field and mobile phone voicemail recordings is illustrated in Fig. 1 .

**Table 1:** Mean and standard deviation of acoustic measures of the free-field and the mobile phone voicemail recordings and p values

	Reference threshold values	Free field (mean ± SD)	Mobile phone (mean ± SD)	p*
*Fundamental frequency parameter*				
**Fo (Hz)**	85–155 (male)	126,73 ± 25,62	126,45 ± 25,67	0,313
*Frequency perturbation parameters*				
**PPQ (%)**	< 0.5	0,35 ± 0,19	0,47 ± 0,29	0,018
**RAP (%)**	< 0.5	0,34 ± 0,21	0,46 ± 0,29	0,020
*Amplitude perturbation parameters*				
**ShdB (dB)**	< 0.5	0,23 ± 0,08	0,54 ± 0,11	<0,001
**Shim (%)**	< 3.0	2,64 ± 0,95	6,19 ± 1,28	<0,001
**APQ (%)**	< 3.5	2,04 ± 0,68	4,96 ± 0,98	<0,001
*Noise parameter*				
**NHR**	< 0.2	0,13 ± 0,02	0,16 ± 0,02	<0,001

Abbreviations: APQ, amplitude perturbation quotient; Fo, average fundamental frequency; NHR, noise-to-harmonic ratio; PPQ, pitch period perturbation quotient; RAP, relative average perturbation; SD, Standard deviation; ShdB, shimmer in dB; Shim, shimmer percent.

* p: Paired *t* -test.

## Discussion

While a few previous studies have scrutinized the quality of voice recordings made with mobile phones, the focus on those transmitted to the other party has been lacking. This limitation has been improved in the current research to some extent. This study aims to provide a detailed investigation of the objective acoustic changes induced by mobile phones specifically in voicemail transmission, focusing on the analysis of the transmitted voice as received by the other party, a topic that has not been extensively covered in existing research. Since the audio file is transmitted identically to the receiving party, our primary focus is on the modifications made by the mobile phone and voicemail application to facilitate voice transmission, particularly app-specific encoding. Analysis of our speaker data revealed that the fundamental frequency remained resistant to alterations in voice; however, all other measures of voice parameters were statistically significantly impacted in voicemail transmissions caused by mobile phones. Our findings share similarities with those of Maryn et al in certain aspects. 8 They found that “fundamental frequency was resistant to the recording system and environmental noise, while all other measures were impacted by both the recording system and noise condition.” In contrast, Uloza et al. demonstrated that “measurements of acoustic voice parameters using smartphones proved to be reliable in clinical settings” 9 . Grillo et al. also showed that there was no significant variability among voice measures with different smartphone recordings. However, their study also included recordings from a head-mounted microphone, which is important because it offers a more standardized recording, minimizing external noise. 16 Petrizzo et al., in their review of six studies, concluded that “smartphones and mobile apps have the potential to be valuable tools in voice assessment outside the clinic” 10 .

Our findings indicate that mobile phones cause significant acoustic changes in voicemail transmission, particularly in frequency perturbation and amplitude perturbation parameters. While these changes are evident, further research is needed to understand their impact on voice quality assessment and clinical applications. Comprehension of spoken messages involves decoding formant information and articulatory acoustics, as well as determining fundamental frequency and overall voice quality for speaker identification. Although the fundamental frequency, which remains resistant to recording and voice transmission, may play a role in understanding the speaker's identity, relying solely on this parameter is unlikely to fulfill this purpose. Therefore, future studies should explore how these mobile phone-induced acoustic changes affect the broader aspects of voice perception and speaker recognition.

Beyond objective changes, mobile phones may also induce perceptual alterations in sound transmission. Passetti et al. 15 concluded in their study that the acoustic quality of mobile phones impacts accurate perception and evaluation of voice quality. They further suggested that these alterations could be relevant in a forensic context. Consequently, the objective changes in human voice over the phone, investigated in this study, might also bear significance for forensic issues.

Examining the effects of certain unavoidable technical limitations in this study, detailing the measures taken to address them, and exploring their impact on the results may contribute to a more nuanced interpretation of the study outcomes. First, measuring real-time voice transmissions as received on another device could introduce numerous unknown and uncontrollable factors. Hence, voicemails were analyzed as an alternative, with the assumption that this method could minimize the variability of factors. Second, the study focused solely on voice parameters and did not assess speech. Nevertheless, it is acknowledged that reducing loudness or eliminating high frequencies from a signal can impact speech intelligibility, particularly affecting consonants. Third, data collection occurred under highly favorable conditions, with voice samples recorded in a soundproof and anechoic room, featuring subjects with normal voices for standardization purposes. However, mobile phones are commonly used in environments with elevated background noise. Lastly, voice transmission across different telephone networks and cellular phone brands may exhibit variability. Additionally, a significant limitation of this study is the use of the Shure SM58 microphone. This dynamic microphone, while suitable for live vocal performance, has a non-flat frequency response (midrange boost and low/high-frequency roll-off) and a slower transient response compared with condenser microphones. Its limited high-frequency range (50 Hz–15 kHz) may have significantly affected the acoustic analysis, particularly in capturing breathiness, roughness, and harmonic-to-noise ratio. We acknowledge the possibility of selection bias inherent in choosing the phonation with the most optimal acoustic measurements. However, to isolate and specifically examine the effects of mobile phone transmission on voice signals, this selection process was deemed essential. Considering these constraints, it is conceivable that sound transmitted over the phone could be further distorted in real-world environments. Furthermore, we suggest that future studies investigate 'Cepstral Peak Prominence' measures, as they are more robust in acoustic voice analysis.

## Conclusion

The current findings suggest that mobile phones induce notable acoustic changes in voicemail transmission. Notably, the fundamental frequency remained resistant to alterations in voice. These results underscore the importance of further research to evaluate the effects of these acoustic changes on voice quality assessment, speaker identification, and clinical applications. Moreover, given the study's technical limitations, future research should investigate real-world conditions to gain a more comprehensive understanding of mobile phone-induced acoustic modifications.
